# CT volumetric analysis: association of renal parenchyma and GFR alteration in nephrectomy patients

**DOI:** 10.1007/s00261-024-04693-y

**Published:** 2024-12-06

**Authors:** Wasin Saengthongpithak, Chalairat Suk-Ouichai, Tawatchai Taweemonkongsap, Shanigarn Thiravit, Ekkarin Chotikawanich, Siros Jitpraphai, Varat Woranisarakul, Katunyou Mahamongkol, Thitipat Hansomwong

**Affiliations:** 1 Division of Urology, Department of Surgery, Faculty of Medicine Siriraj Hospital, Mahidol University, Bangkok, Thailand; 2 Division of Diagnostic Radiology, Department of Radiology, Faculty of Medicine Siriraj Hospital, Mahidol University, Bangkok, Thailand

## Background

Renal parenchyma volume (RPV) and nephron mass are essential indicators of renal function. A reduction in nephron mass and RPV is commonly observed during renal senescence and in patients with chronic kidney disease (CKD) and is especially prominent following nephrectomy. While the long-term impact of surgically induced CKD in the general population, such as in living kidney donors, remains uncertain, studies have highlighted the detrimental effects of renal parenchyma loss following treatment for renal cell carcinoma (RCC).

CT scanning is the standard modality for renal tumor diagnosis, surgical planning, and early follow-up. It also serves as a primary evaluation tool for kidney donors [1]. With the advent of RPV measurement or CT volumetry, clinicians can now estimate residual RPV following ablative therapy or nephrectomy. This approach enables individualized counseling on CKD risk and more accurate predictions of postoperative renal function or glomerular filtration rate (GFR). Recent studies have demonstrated that contrast-enhanced CT scans can predict new baseline GFR (NB-GFR) following radical and donor nephrectomy with accuracy that is non-inferior or even superior to traditional MAG-3 renal scintigraphy. Although alternative, radiation-free imaging technologies are emerging for preoperative evaluation, CT remains the primary choice due to its detailed imaging capability and established clinical utility [1–4]. Furthermore, CT volumetry overcomes limitations of nuclear renal scans in partial nephrectomy (PN) cases.

Nephrectomy remains a cornerstone in RCC treatment, delivering favorable oncological outcomes. Nephron-sparing procedures, such as PN and focal tumor ablation, preserve more RPV while achieving comparable oncological results [5]. However, a reduction in GFR is often unavoidable, leading to an increased risk of hospitalization, cardiovascular morbidity [6], and limitation on available chemotherapy or targeted therapy options for patients with reduced GFR [7]. Additionally, patients with NB-GFR values below 45 mL/min/1.73 m² face significant higher risk of non-cancer-related mortality [8–10].

Several novel GFR prediction models have been introduced [11–13]. Palacios et al. developed an NB-GFR prediction model for partial and radical nephrectomy patients, incorporating patient characteristics and demographic data, which has been validated with high accuracy in external studies [12, 14]. Similarly, van Londen et al. developed a NB-GFR prediction model specifically for living kidney transplant donors [14]. This highlights the necessity of incorporating accurate GFR predictions into treatment decisions.

This study aims to investigate RPV changes following different types of nephrectomies, explore the correlation between altered parenchyma volume and actual NB-GFR, identify factors associated with NB-GFR, and evaluate the ability of CT volumetry to predict NB-GFR.

## Methods

### Study population

This is a single-center retrospective cohort study. Subjects were recruited from a single-center database between January 2017 and December 2021, including RCC patients aged 18 years or older who underwent partial or radical nephrectomy (RN) via either open or minimal invasive approaches at Siriraj hospital. Patients with resectable tumors, including those with locally advanced stages or renal vein thrombus, were included. In the donor nephrectomy (DN) group, we included living kidney donors who underwent DN at our institute during the period. Exclusion criteria were age below 18 years, congenital or anatomical kidney anomalies, synchronous multiple renal tumors, and preoperative GFR < 15 mL/min/1.73 m².

All patients in this study had a preoperative GFR measured using the CKD-EPI formula from samples collected one day before surgery, as well as a preoperative contrast-enhanced CT scan with a slice thickness of 3 mm or less. For patients with borderline pre-CT GFR (< 45 mL/min/1.73 m²), a multidisciplinary approach involving nephrologists was used to implement preventive measure against contrast-induced nephropathy. None of the enrolled patients experienced contrast-induced nephropathy before surgery. For postoperative data, the first GFR reading collected between 3 and 12 months postoperatively and a contrast-enhanced CT scan within the same period were required for RCC patients. For kidney donors, the postoperative GFR recorded at the first transplant clinic visit was used as a reference.

### Volumetric analysis

**Fig. 1:**
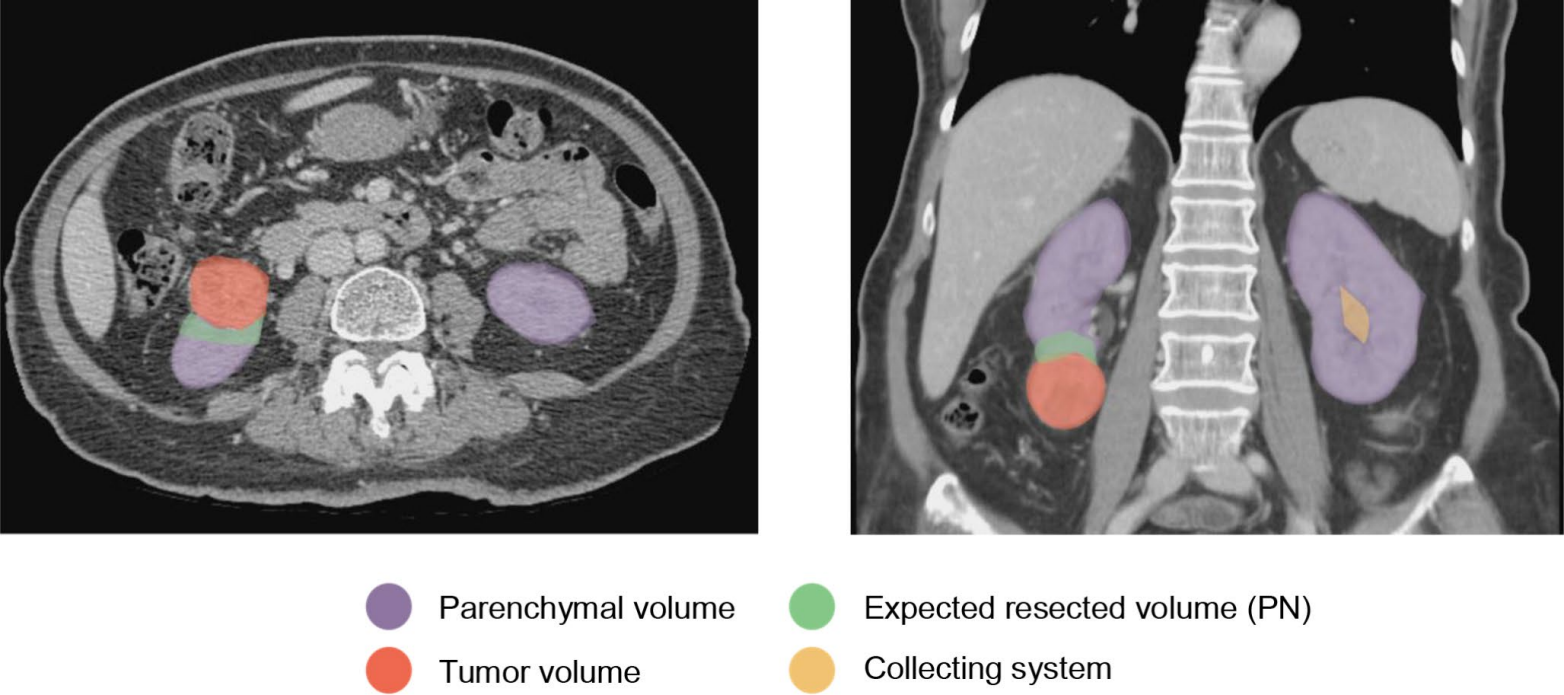
Outline of volume derived from volumetric analysis from a patient with a lower pole renal cell carcinoma

**Fig. 2:**
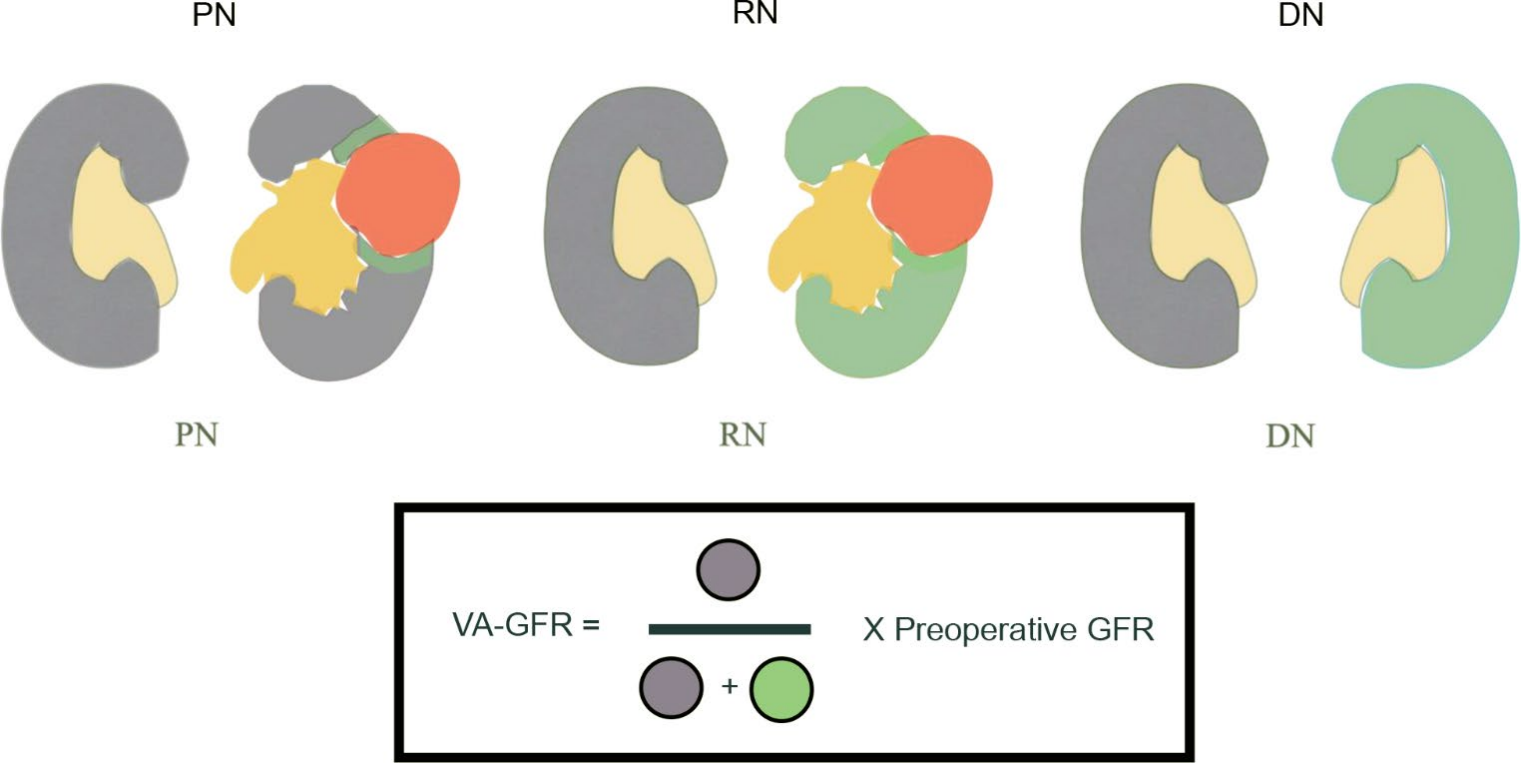
Volumetric analysis on different types of nephrectomy and sample volume calculation of VA-GFR from derived volume

**Fig. 3:**
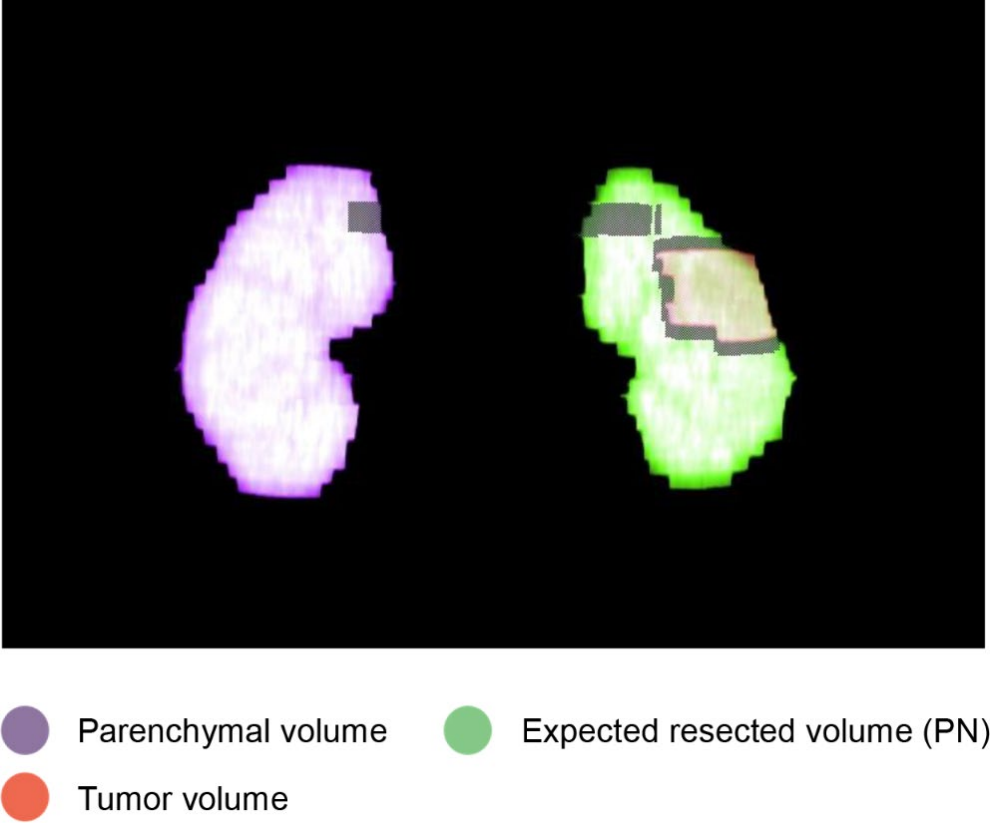
Three - dimensional rendering images of CT volumetric analysis from a radical nephrectomy case. The colors representing different volumes were demonstrated as labeled

Volumetric analysis in this study refers to the calculating the volume (PV, cm^3^) of the renal parenchyma, tumor, excised parenchyma, and remaining parenchyma. A contrasted-enhanced CT scan with a slice thickness of 3 mm or less in the axial plane was utilized. The kidney with the tumor or selected for DN was termed the resected side, while contralateral kidney was defined as the preserved side. Volume measurements were obtained using freehand, slide-by-slide drawing with a third-party application (GE workstation; GE healthcare Inc., IL, USA). The volume was calculated by multiplying the identified area by the slide thickness. Two urology residents performed the volumetric analysis under the close supervision of a board-certified urologist and a radiologist. Figures 1, 2 and 3 display data derived from volumetric analysis.

In preoperative imaging, once total RPV was identified, resected PV was estimated individually. For RN and DN cases, the resected PV represented the entire kidney to be removed or the kidney on the tumor’s side. In PN cases, resected PV were estimated with a 1 cm margin around the renal mass. VA-GFR was calculated as: “VA-GFR = ((Total preoperative RPV– Predicted resected PV)/Total PV) x Preoperative GFR”. Preoperative GFR per volume was calculated as: “Preoperative GFR per volume = Preoperative GFR/ Total preoperative RPV”.

In postoperative imaging, remaining PV and postoperative GFR per volume were calculated in a similar manner. Split and total PV compensation were defined as: “% PV compensation = ((Postoperative PV– Preoperative PV)/Preoperative PV) x 100”.

### Data analysis

For demographic data, nominal variables were reported as frequencies and percentages, while continuous variables were presented as means or medians and interquartile ranges.

To assess the correlations between VA-GFR and actual NB-GFR, Pearson correlation coefficients were calculated for each subgroup. A higher correlation coefficient indicates a stronger correlation, with strong and very strong correlations defined as *r* > 0.7 and > 0.9, respectively.

For volumetric analysis, the Intraclass Correlation Coefficient (ICC) was used to validate interrater reliability on a sample data set, with good and excellent reliability defined as ICC > 0.75 and > 0.9, respectively. A subgroup analysis based on the types of nephrectomy (PN, RN, and DN) was conducted to assess differences between groups. One-way ANOVA was used to identify any statistical differences among groups.

For univariate analysis, different tests were applied based on the data type. The Chi-Square test and Wilcoxon test were employed to compare nominal variables, while an independent t-test and linear regression analysis were used to compare continuous variables between groups. Multivariate analysis was performed using linear regression. A p-value < 0.05 was considered statistically significant.

The interrater reliability of the novel NB-GFR prediction model, compared to the Palacios and van Londen models, was evaluated using Cohen’s Kappa coefficient. Model agreement was considered substantial when the coefficient was > 0.6 and almost perfect when > 0.8. The same patient population was used to evaluate the model’s sensitivity, specificity, and accuracy in predicting NB-GFR < 45 mL/min/1.73 m², in comparison with both models.

## Results

The demographic data of the patients included in this study are shown in Table 1. The median age in the PN group was 63 years, while it was 60 years in the RN group. The majority of RCC patients were male in both groups (72.5% in the PN group and 70% in the RN group). In contrast, there was a significant female predominance (58%) and a lower median age (35 years) in the DN group. BMI was significantly higher in PN group and lowest in the RN group.

**Table 1:** Demographic data

	PN (*n* = 40)	RN (*n* = 40)	DN (*n* = 38)	*p* value
Age (Median, IQR)	63(53–73)	60 (51–69)	35 (29–46)	< 0.001 (PN vs. DN)
Gender (Male, %)	29 (72.5)	28 (70)	16 (42.1)	0.016 (PN vs. DN)
Hypertension (Yes, %)	29 (72.5)	22 (55)	0	< 0.001 (PN vs. DN)
Diabetes mellitus (Yes, %)	16 (40)	12 (30)	0	< 0.001 (PN vs. DN)
Preoperative proteinuria (Yes, %)	6 (15)	10 (25)	1 (2.6)	0.34
Postoperative proteinuria (Yes, %)	9 (22.5)	12 (30)	4 (10.5)	0.5
Body mass index (kg/m^2^, Median, IQR)	25.55 (24.5–29)	22.9 (21–25)	24.24 (20–25)	< 0.001 (PN vs. RN, DN)
Preoperative chronic kidney disease stage (%)				< 0.001 (PN vs. DN)
Grade 1 eGFR 90 ml/min/1.73 m^2^ or greater	7 (17.5)	14 (35)	35 (92.1)	
Grade 2 eGFR 60–89 ml/min/1.73 m^2^	23 (57.5)	16 (40)	3 (7.9)	
Grade 3a eGFR 45–59 ml/min/1.73 m^2^	10 (25)	8 (20)	0	
Grade 3b eGFR 30–44 ml/min/1.73 m^2^	0	2 (5)	0	
Grade 4 eGFR 15–29 ml/min/1.73 m^2^	0	0	0	
Preoperative CT to operative date duration (Days, Median, IQR)	53.5 (29–88)	46 (27–73.5)	182.5 (125–268)	< 0.001 (PN vs. DN)
Operative date to postoperative CT duration (Days, Median, IQR)	189 (130.8–291)	141 (107.5–220)	N/A	0.57
Nephrectomy side (Left, %)	25 (62.5)	20 (50)	31 (81.6)	0.22
Surgical approach (n, %)				< 0.001 (PN vs. RN, RN vs. DN)
Open	19 (47.5)	33 (82.5)	8 (21.1)	
Laparoscopy	1 (2.5)	6 (15)	27 (71)	
Robotic-assisted laparoscopy	20 (50)	1 (2.5)	3 (7.9)	
Ischemia type (n, %)				
Zero ischemia	7 (17.5)	N/A	N/A	
Cold ischemia	10 (25)	N/A	N/A	
Warm ischemia	23 (57.5)	N/A	N/A	
Tumor diameter (cm., Median, IQR)	3.5 (2.1–4.5)	9 (6.63–13)	0	< 0.001 (PN vs. RN, DN)
Cell type				
Clear cell (n, %)	35 (87.5)	29 (72.5)	0	
Papillary type 1 (n, %)	2 (5)	2 (5)	0	
Papillary type 2 (n, %)	2 (5)	3 (7.5)	0	
Chromophobe (n, %)	1 (2.5)	5 (12.5)	0	
Undifferentiated (n, %)	0	1 (2.5)	0	

IQR interquartile range; GFR glomerular filtration rate; PN partial nephrectomy; RN Radical nephrectomy; DN Donor nephrectomy

The comorbidities included in this study were hypertension (72.5% in the PN vs. 55% in RN), diabetes mellitus (40% in the PN and 30% in the RN), and preoperative proteinuria (15% in the PN vs. 25% in the RN). Tumor diameter was significantly larger in the RN group compared to the PN group (9 cm vs. 3.5 cm, respectively). Most tumors were of the clear cell type (87.5% in the PN and 72.5% in the RN). In the DN group, there were no records of hypertension or diabetes mellitus, and only one patient (2.6%) had preoperative proteinuria. No patient had a preoperative GFR of less than 30 ml/min/1.73 m^2^.

In the PN group, 20 patients underwent robotic-assisted PN, one patient underwent laparoscopic PN, and 19 patients had open PN. Renal artery clamping without surface cooling was used in 23 patients, while 10 patients received surface cooling (cold ischemia), and 7 patients underwent PN without any ischemic period.

**Fig. 4:**
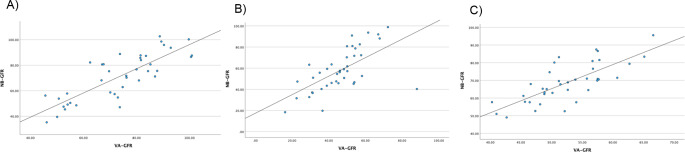
Scatter dot plot demonstrating subgroup correlation between NB-GFR and VA-GFR in (**A**) PN (**B**) RN and (**C**) DN group. PN Partial nephrectomy; RN Radical nephrectomy; DN Donor nephrectomy; NB-GFR New baseline glomerular filtration rate; VA-GFR Volumetric analysis predictive new baseline GFR

Table 2 shows the comparison between preoperative eGFR, NB-GFR, predicted new baseline GFR based on the Palacios equation [12], and VA-GFR. The dot plot between NB-GFR VA-GFR is shown on Fig. 4 The Pearson correlation coefficient indicated a strong correlation between NB-GFR and Palacios GFR in all types of nephrectomy (0.789 in PN, 0.756 in RN, and 0.755 in the DN group, all with *p* < 0.001). Notably, VA-GFR showed the highest correlation with NB-GFR for patients who underwent PN (*r* = 0.814), while moderate and strong correlations were observed for patients who underwent RN and DN (*r* = 0.625 and 0.759, respectively).

**Table 2:** GFR comparison between VA-GFR, NB-GFR, and Palacios GFR

	PN (*n* = 40)	RN (*n* = 40)	DN (*n* = 38)
Preoperative GFR (ml/min/1.73 m^2^, Median, IQR)	77 (60–87)	82.49 (61–95)	106.67 (99–112)
VA-GFR (ml/min/1.73 m^2^, Median, IQR)	74.27 (58–85)	47.15 (36–53)	51.79 (48–57)
Palacios GFR (ml/min/1.73 m^2^, Median, IQR)	69.83 (56–77)	60.52 (42–66)	76.6 (72–82)
NB-GFR (ml/min/1.73 m^2^, Median, IQR)	73.51 (55–86)	55 (45–72)	68.37 (60–77)
Remaining GFR (%, Median, IQR)	92.06 (82–104)	72.48 (61–87)	64.7 (59–68)
Pearson Correlation ^®^ between VA-GFR & NB-GFR	0.814 (*p* < 0.001)	0.625 (*p* < 0.001)	0.759 (*p* < 0.001)
Pearson Correlation ^®^ between Palacios & actual GFR	0.789 (*p* < 0.001)	0.756 (*p* < 0.001)	0.755 (*p* < 0.001)

IQR interquartile range; PN partial nephrectomy; RN radical nephrectomy; DN donor nephrectomy; PV parenchymal volume; RPV remaining parenchymal volume; NB-GFR new baseline glomerular filtration rate; VA-GFR volumetric analysis predictive new baseline GFR; VA-GFR calculation was ((total preoperative RPV– predicted resected PV)/Total PV) x preoperative GFR)

Table 3 presents findings derived from the volumetric analysis. The total preoperative PV was significantly lower in the DN group (260 cm^3^) compared to patients with RCC. When focusing on the PV of the kidney with RCC, the PV was significantly larger in the PN group (149.34 cm^3^ vs. 115.89 cm^3^). The mean preoperative PV of “preserved side” was similar in the PN and RN groups while it was lower in living kidney donors (154.49 cm^3^, 161.21 cm^3^, and 127.15 cm^3^, respectively). The difference was significant when comparing the PN and DN groups (*p* < 0.001). The mean tumor volume was significantly smaller in the PN group compared to the RN group (21.31 vs. 382.04 cm^3^, *p* < 0.001).

**Table 3:** Compensation in volumetric analysis

		PN (*n* = 40)	RN (*n* = 40)	DN (*n* = 38)	*p* value
Preoperative parenchyma volume (cm^3^, Median, IQR)	Resected side	149.34 (129–176)	115.89 (96–152)	133.41 (84–205)	< 0.001 (PN vs. RN)
	Preserved side	154.49 (145–170)	161.21 (138–186)	127.15 (112–145)	< 0.001 (DN vs. RN, PN)
	Total	307.21 (267–341)	287.67 (242–339)	260 (232–297)	0.016 (PN vs. DN)
Postoperative parenchyma volume (cm^3^, Median, IQR)	Resected side	120.99 (103–140)	0	N/A	< 0.001
	Preserved side	164.12 (142–181)	180.54 (154–202)	N/A	< 0.001
	Total	278.35 (254–320)	180.54 (154–202)	N/A	< 0.001
Volume compensation (%, Median, IQR)	Resected side	-17.9 (-49–10)	0	N/A	< 0.001
	Preserved side	2.8 (-1–7)	12.86 (-2–19)	N/A	0.004
Preoperative GFR per volume ((ml/min/1.73 m^2^)/cm^3^, Median, IQR)		0.25 (0.19–0.28)	0.29 (0.22–0.35)	0.4 (0.32–0.49)	< 0.001 (PN vs. DN)
Postoperative GFR per volume ((ml/min/1.73 m^2^)/cm^3^, Median, IQR)		0.24 (0.2–0.3)	0.35 (0.24–0.41)	N/A	< 0.001 (PN vs. RN)
GFR per volume compensation (%, Median, IQR)		3.89 (-10–14)	12.95 (3–19)	N/A	0.006 (PN vs. RN)
NB-GFR < 45 ml/min/1.73 m^2^ (n, %)		2 (5)	9 (22.5)	0	

IQR interquartile range; PN partial nephrectomy; RN Radical nephrectomy; DN Donor nephrectomy; GFR glomerular filtration rate; NB-GFR = new baseline GFR

In the postoperative period, for the kidneys not affected by the surgery, the remaining kidney volume was significantly larger in the RN group (182.52 cm^3^ vs. 161.38 cm^3^, *p* < 0.001). This finding correlated with a 12.86% volume compensation of the remaining renal unit within this group. Interestingly, the remaining parenchyma of the resected kidney in the PN group showed no hypertrophic compensation and only a slight volume increase (2.8%) on the unaffected side. However, total postoperative PV was significantly higher in the PN group. Considering the pre- and postoperative eGFR and renal PV, there was a significantly increase in GFR per 1 cm^2^  of renal PV in the RN group (from 0.29 preoperatively to 0.35 postoperatively), representing a 12.95% increase vs. 3.89% increase in the PN group (see Table 3).

Uni- and multivariate analyses of factors affecting poorer NB-GFR are summarized in Table 4. Significant predictors of NB-GFR included advancing age (*p* =0.019), VA-GFR (*p* < 0.001), male gender (*p* < 0.001), hypertension (*p* = 0.05), and preoperative proteinuria (*p* = 0.04).

**Table 4:** Univariate and multivariate analysis on factors affecting NB-GFR

		Univariate analysis		Multivariate analysis	
		Coefficient	*p* value	Coefficient	*p* value
Age*		-0.376	< 0.001	-0.247	0.019
Preoperative GFR*		0.495	< 0.001	-	-
VA-GFR*		0.691	< 0.001	0.777	< 0.001
Male gender^#^		-10.87	< 0.001	-7.849	< 0.001
Hypertension^#^		-10.65	0.002	-5.807	0.05
Diabetes mellitus^#^		-5.92	0.185	-1.192	0.66
Tumor size > 7 cm^#^		-2.15	0.617	-2.746	0.42
Nephrectomy type^#^	PN vs. DN	2.33	1.00	-0.263	0.96
	PN vs. RN	12.963	0.003	4.771	0.26
Preoperative proteinuria^#^		-6.60	0.158	-6.612	0.04

* Linear regression; # Mean difference; NB-GFR = new baseline GFR; VA-GFR volumetric analysis predictive new baseline GFR; PN  Partial nephrectomy; RN  radical nephrectomy; DN  donor nephrectomy

The proposed predictive model for calculating NB-GFR were created, based on a volumetric-based predicted NB-GFR using backward linear regression analysis. The model is as follows:

NB-GFR = 40.08 + 0.79(VA-GFR) − 7.734(if Male) − 6.632(if Hypertension) − 5.954(if Preoperative proteinuria) − 0.249(Age) + 6.726(if Radical nephrectomy) − 0.228(if Donor nephrectomy).

**Fig. 5:**
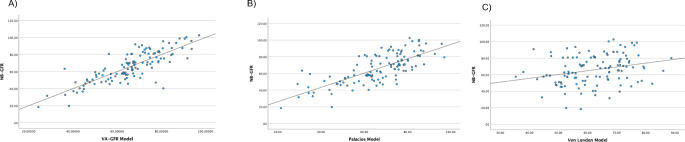
Scatter dot plot demonstrating correlation between (**A**) NB-GFR and VA-GFR Model and (**B**) NB-GFR and Palacios Model and (**C**) NB-GFR and van Londen Model. PN Partial nephrectomy; RN Radical nephrectomy; DN Donor nephrectomy; NB-GFR New baseline glomerular filtration rate; VA-GFR Volumetric analysis predictive new baseline GFR

Using the Palacios RCC model and van Londen DN model as references, As shown on Dot– Plot Fig. 5, the novel GFR prediction model (VA-GFR model) demonstrated substantial interrater reliability with a Cohen’s Kappa coefficient of 0.776 compared to RCC model. In contrast, DN model displayed a different trend in NB-GFR compared to VA-GFR model with Cohen’s Kappa coefficient of 0.166. Table 5 shows the number of patients with NB-GFR < 45 ml/min/1.73 m^2^. Unfortunately, 11 patients (2 in the PN group and 9 in the RN group) were classified as CKD 3b postoperatively. According to VA-GFR model, one patient in the RN group had an unexpected NB-GFR < 45 ml/min/1.73 m^2^. In contrast, based on the RCC model, one patient in the PN group and two patients in the RN group were unexpectedly classified as CKD stage 3b postoperatively. The sensitivity, specificity, and accuracy of VA-GFR model in predicting NB-GFR < 45 ml/min/1.73 m^2^ were 90.9%, 98.1% and 97.5%, respectively. The RCC model demonstrated 72.7% sensitivity, 95.3% specificity, and 93.2% accuracy, while the DN model showed 9.1% sensitivity, 95.3% specificity, and 87.2% accuracy.

**Table 5:** Performance comparison between VA-GFR and other models that utilize preoperative characteristics for NB-GFR prediction NB-GFR = new baseline GFR; VA-GFR volumetric analysis predictive new baseline GFR

	VA-GFR model	Palacios model	van Londen model
Total events of predicted NB-GFR < 45 (n)	12	13	6
Total false positive events (Unexpected NB-GFR > 45) (n)	2	3	4
Total false negative events (Unexpected NB-GFR < 45) (n)	1	2	10
Sensitivity/ Specificity/ Accuracy (%)	90.9/ 98.1/ 97.5	72.7/ 95.3/ 93.2	9.1/ 95.3/ 87.2
Cohen’s Kappa coefficient vs. VA-GFR model	-	0.776	0.166

## Discussion

Accurate prediction of NB-GFR for patients undergoing nephrectomy is essential, offering prognostic benefits for preoperative counseling [6], potentially reducing morbidity, especially cardiovascular events, and expanding eligibility for adjuvant treatments [7, 8, 15]. This study highlights differences in RPV between RCC patients and living kidney donors, with the latter having fewer underlying morbidities. We observed varying RPV compensation patterns and their relationships with NB-GFR across different nephrectomy types, underscoring the value of preoperative RPV measurement from routine CT scans for individualized counseling, especially in patient undergoing PN, where NRS predictability may be limited.

Previous studies have shown that RPV varies based on gender and body habitus [16, 17]. In this study, patients with small renal masses eligible for PN demonstrated the highest preoperative PV, whereas those scheduled for DN had the lowest preoperative PV. These findings suggest that preoperative PV compensation might be influenced by the presence of renal tumors. However, the interpretation of these variations is limited by factors such as the female predominance, younger age, fewer comorbidities in the DN group, and higher BMI in the PN group. Additionally, the specific effects of RCC and its histologic subtypes on preoperative RPV remain unclear, potentially limiting volumetric analysis conclusions.

For surgeons managing complex renal masses, particularly in patients with limited preoperative GFR or hereditary conditions that predispose them to multiple tumors, accurately predicting NB-GFR or CKD risk is crucial. Studies indicate that patients undergoing nephrectomy typically experience a 20–30% reduction in renal function [18–20], while those undergoing nephron-sparing surgery maintain 90–95% of their renal function postoperatively [12, 19]. Previous reports have shown that split PV-based models, incorporating remaining PV, preoperative GFR, and a 25% compensation factor, can predict NB-GFR effectively [18]. In this study, VA-GFR, derived from preoperative RPV, showed the strongest correlation with NB-GFR in the PN group and a high correlation in the DN group. Compared to models relying solely on preoperative characteristics, PV analysis offers improved performance in predicting NB-GFR, especially in PN cases. Expected resected parenchyma helps guide the surgical approach, minimizing parenchymal loss and preserving NB-GFR. However, data on PV analysis accuracy for obstructed kidneys or those with renal vein or inferior vena cava thrombus are limited. Previous studies have shown that conditions like hydronephrosis, pyelonephritis, and renal vein thrombosis alter PV analysis [21]. Additionally, the impact of various treatment modalities, including tumor ablation, laparoscopic or robotic-assisted approaches, and renal vascular clamping duration (including parenchymal cooling), on RPV and NB-GFR predictive accuracy requires further study [2, 22, 23].

This study focused on changes in NB-GFR and RPV within the first year post-nephrectomy, as long-term GFR and RPV could be influenced more by patient characteristics or progression of underlying conditions than by nephrectomy itself. In contrast, previous studies have suggested that GFR recovery may continue for up to 25 months post-nephrectomy, with compensatory hypertrophy frequently observed [24]. The hyperfiltration theory has been proposed to explain this phenomenon [25]. The degree of compensation varies and depends largely on preoperative parenchymal and glomerular health. Greater compensatory hypertrophy is associated with less preoperative fibrosis and glomerular injury, underscoring the importance of considering underlying conditions and patient characteristics when predicting postoperative RPV and GFR changes. In our study, kidneys that underwent PN did not show hypertrophic PV compensation post-surgery, while the contralateral kidney exhibited a slight PV increase. Interestingly, in RN cases where preoperative hypertrophic compensation was suspected, the preserved kidney still showed the greatest PV increase postoperatively. Moreover, GFR per cubic centimeter of RPV was significantly higher in RN cases compared to PN cases, highlighting the need for preoperative evaluation of underlying conditions that could impact compensatory capacity.

Consistent with findings from larger studies [12, 14], this study found that baseline characteristics, including advancing age, male gender, hypertension, proteinuria, preoperative eGFR, and remaining PV percentage, significantly impacted NB-GFR. By incorporating these factors with VA-GFR, we propose a novel NB-GFR prediction model based on CT volumetry that may generalize to all nephrectomy types. The VA-GFR model demonstrated substantial interrater reliability and promising sensitivity, specificity, and accuracy compared to the RCC and DN models [12, 15]. Given the critical GFR cutoff of 45 mL/min/1.73 m², VA-GFR models accurately predicted de novo CKD with 97.5% accuracy following nephrectomy. A few patients, however, presented with unexpectedly low NB-GFR (< 45 mL/min/1.73 m²). Despite reviewing patient histories and operative findings, no clear cause was identified for these declines, highlighting the limitations of existing tools in accurately predicting significant renal function loss. Combining the VA-GFR model with other predictive tools, such as the RENSAFE AKI and CKD nomogram [26] for RN cases or Cystatin C [27] in DN cases, may help reduce the risk of unexpected low NB-GFR occurrences.

This study has several limitations. First, the retrospective design may introduce selection bias in treatment choices and surgical approaches, though volumetric analysis was derived from the least parenchymal resection possible. Second, manual volumetric analysis is time-intensive compared to earlier methods, such as contact surface area or spherical excised parenchymal prediction methods [28, 29]. Automated or semi-automated RPV calculations have shown accuracy [30–32], but their validation in PN cases is needed. Third, the limited follow-up period restricts assessment, as NB-GFR is a single point-in-time outcome that does not ensure long-term renal function preservation, which may be influenced by multiple factors. Nevertheless, recent study has shown a correlation between CT-predicted and actual NB-GFR over time [24]. Fourth, cases with renal anomalies, ureteral obstruction, or previous diversion were excluded. Fifth, the study population was limited to Thai patients. Recent studies show varying post-nephrectomy CKD incidences across races [33], highlighting the need for external population validation to assess the model’s generalizability. Finally, contrast-enhanced CT scans can impact renal function, particularly in CKD patients, underscoring the need for future studies on non-contrast and non-irradiating imaging techniques for RPV assessment.

Despite these limitations, our study provides a foundation for more accurate predictions of NB-GFR in patients undergoing nephrectomy.

## Conclusion

Renal function decline following nephrectomy is associated with reduced survival. CT volumetric analysis has identified patterns of renal parenchymal alteration across different nephrectomy types. This study shows a strong correlation between VA-GFR and actual NB-GFR, especially in patients undergoing PN. Our VA-GFR-based model demonstrates improved accuracy in NB-GFR prediction across all nephrectomy types, surpassing previous models. Integrating volumetric analysis into preoperative assessments enhances outcome prediction, offers prognostic benefits, and ultimately supports long-term, personalized care for post-nephrectomy patients.

## Electronic supplementary material

Below is the link to the electronic supplementary material.

Supplementary Material 1

Supplementary Material 2

Supplementary Material 3

## Data Availability

Data is available upon request from TH for academic purposes.
