# Supplementary material for: CT volumetric analysis: association of renal parenchyma and GFR alteration in nephrectomy patients
Source: Abdom Radiol (NY). 2024 Dec 6;50(6):2549–57. doi: 10.1007/s00261-024-04693-y (PMC12069147; doi:10.1007/s00261-024-04693-y)
Supplement: Supplementary file 1 — Supplementary Material 1 [file 261_2024_4693_MOESM1_ESM.docx]

**Purpose:**

To investigate changes in renal parenchyma volume (PV) following different types of nephrectomy, their correlation with new baseline GFR (NB-GFR), and associated factors.

**Methods:**

A total of 118 patients who underwent partial (PN), radical (RN), or donor (DN) nephrectomy were included. CT volumetric analysis (VA) was used to measure preoperative, predicted, and actual post-nephrectomy PV. Predicted NB-GFR (VA-GFR) was calculated based on estimated remaining PV from preoperative VA. The Pearson correlation was used to assess the relationship between actual NB-GFR and VA-GFR. Linear regression identified predictors of actual NB-GFR.

**Results:**

Of the 118 patients, 40 underwent PN, 40 underwent RN, and 38 underwent DN, respectively. Median preoperative GFRs were 77, 82.5, and 106.7 for PN, RN, and DN, respectively, with corresponding preoperative PVs of 307.2 ml, 287.7 ml, and 260 ml. Percentages of remaining GFR were 92.1% (PN), 72.5% (RN), and 64.7% (DN), while remaining total PVs were 90.6% (PN) and 62.8% (RN). Postoperative PV increases in the non-operated kidney were greater in RN (12.9%) than in PN (2.8%). VA-GFR showed strong correlations with actual NB-GFR in RN (r = 0.63) and DN (r = 0.76), and a very strong correlation in PN (r = 0.81). Multivariate analysis revealed that age, male gender, preoperative GFR, VA-GFR, and preoperative proteinuria were significantly associated with NB-GFR.

**Conclusion:**

VA-GFR has demonstrated a high correlation with actual NB-GFR within one year of nephrectomy. CT VA identified distinct patterns of renal parenchymal changes across nephrectomy types. Integrating volumetric analysis into preoperative evaluations may improve outcome prediction, ultimately improving patient care and prognosis.
