# Supplementary material for: CT volumetric analysis: association of renal parenchyma and GFR alteration in nephrectomy patients
Source: Abdom Radiol (NY). 2024 Dec 6;50(6):2549–57. doi: 10.1007/s00261-024-04693-y (PMC12069147; doi:10.1007/s00261-024-04693-y)
Supplement: Supplementary file 3 — Supplementary Material 3 [file 261_2024_4693_MOESM3_ESM.pptx]

## Slide 1
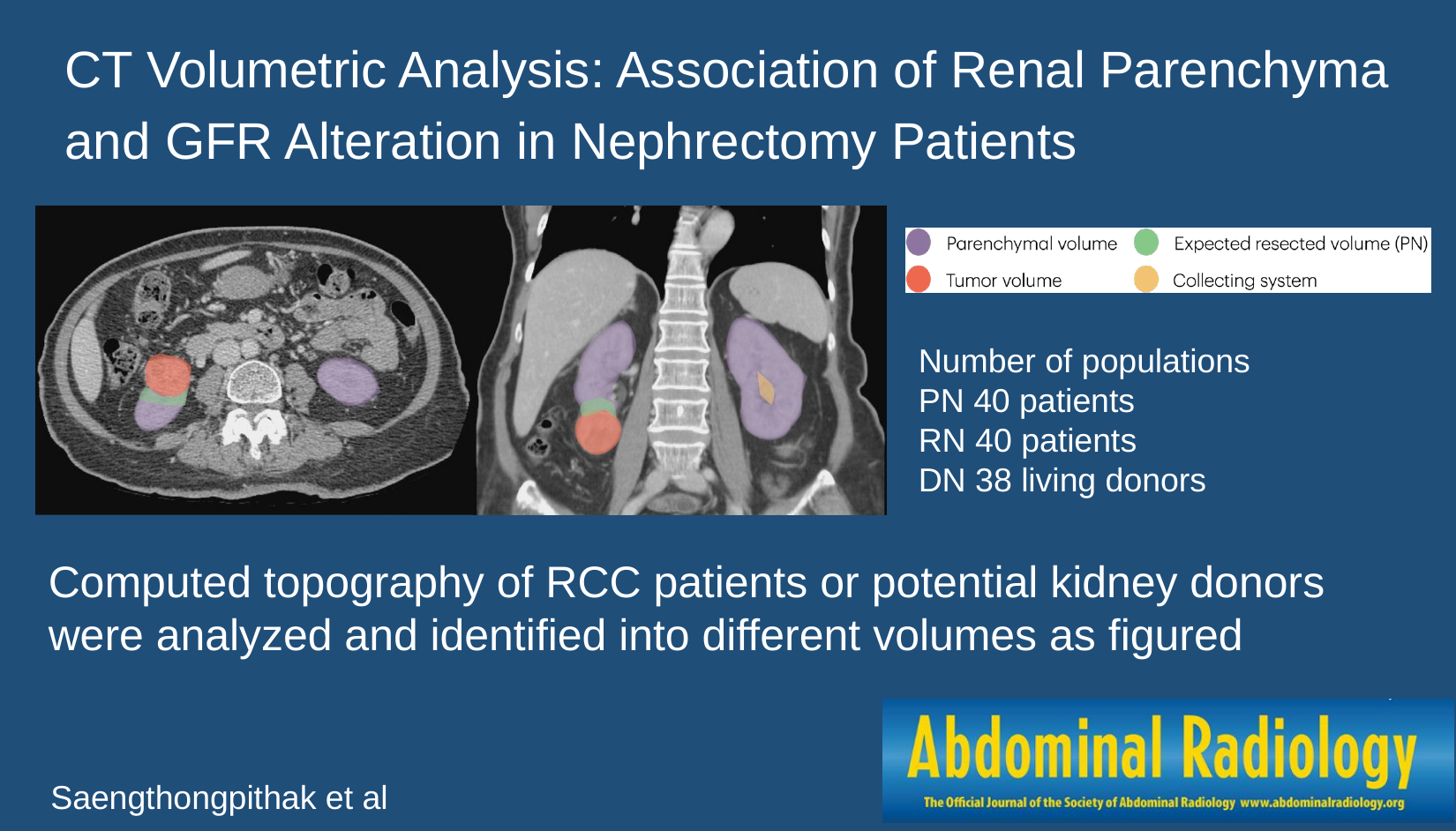

CT Volumetric Analysis: Association of Renal Parenchyma and GFR Alteration in Nephrectomy Patients
Number of populations
PN 40 patients
RN 40 patients
DN 38 living donors
Computed topography of RCC patients or potential kidney donors were analyzed and identified into different volumes as figured
Saengthongpithak et al

## Slide 2
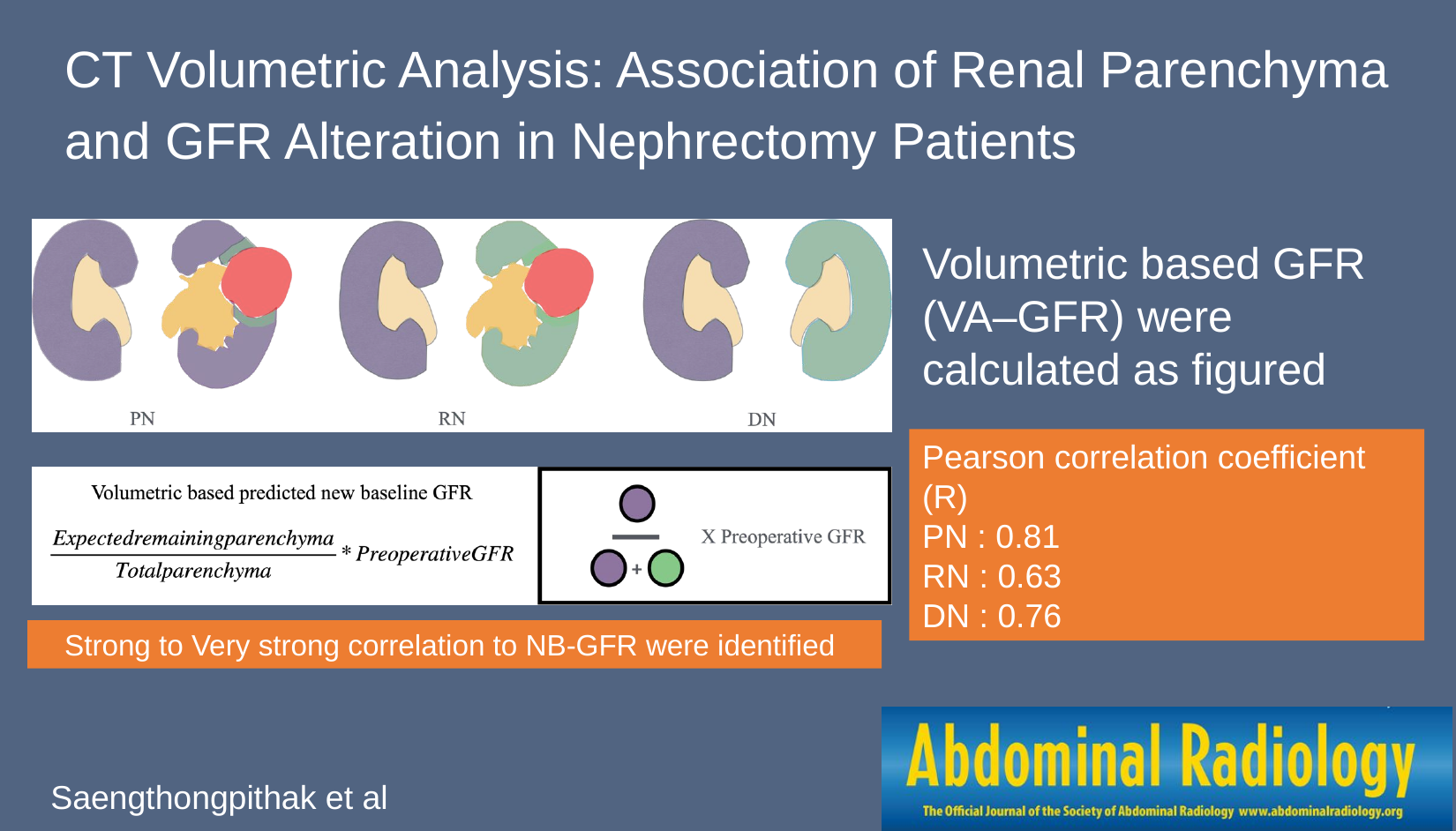

CT Volumetric Analysis: Association of Renal Parenchyma and GFR Alteration in Nephrectomy Patients
Volumetric based GFR
(VA–GFR) were calculated as figured
Pearson correlation coefficient (R)
PN : 0.81
RN : 0.63
DN : 0.76
Strong to Very strong correlation to NB-GFR were identified
Saengthongpithak et al

## Slide 3
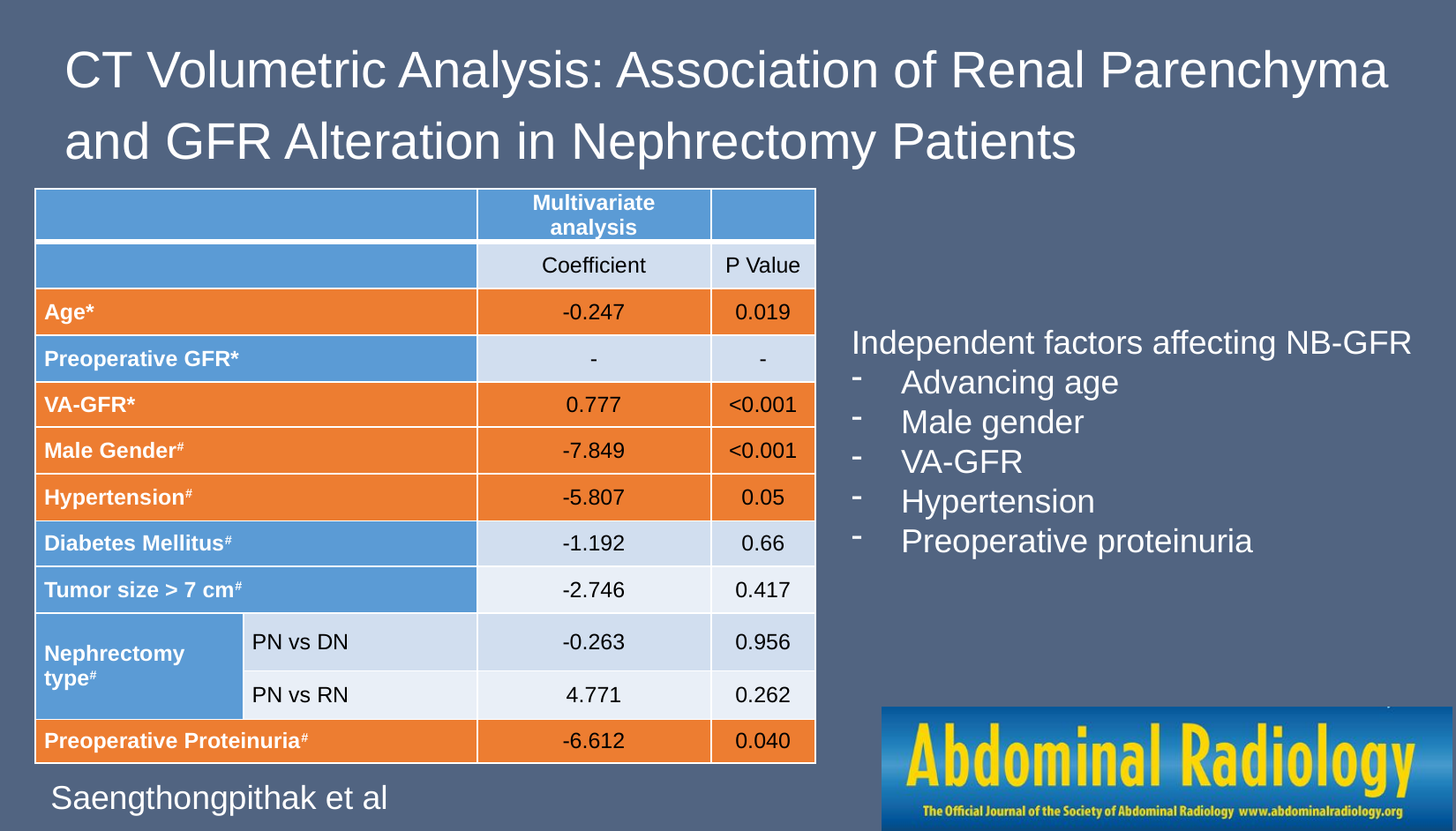

CT Volumetric Analysis: Association of Renal Parenchyma and GFR Alteration in Nephrectomy Patients
| | | Multivariate analysis | |
| --- | --- | --- | --- |
| | | Coefficient | P Value |
| Age\* | | -0.247 | 0.019 |
| Preoperative GFR\* | | - | - |
| VA-GFR\* | | 0.777 | <0.001 |
| Male Gender# | | -7.849 | <0.001 |
| Hypertension# | | -5.807 | 0.05 |
| Diabetes Mellitus# | | -1.192 | 0.66 |
| Tumor size > 7 cm# | | -2.746 | 0.417 |
| Nephrectomy type# | PN vs DN | -0.263 | 0.956 |
| | PN vs RN | 4.771 | 0.262 |
| Preoperative Proteinuria# | | -6.612 | 0.040 |
Independent factors affecting NB-GFR
Advancing age
Male gender
VA-GFR
Hypertension
Preoperative proteinuria
Saengthongpithak et al

## Slide 4
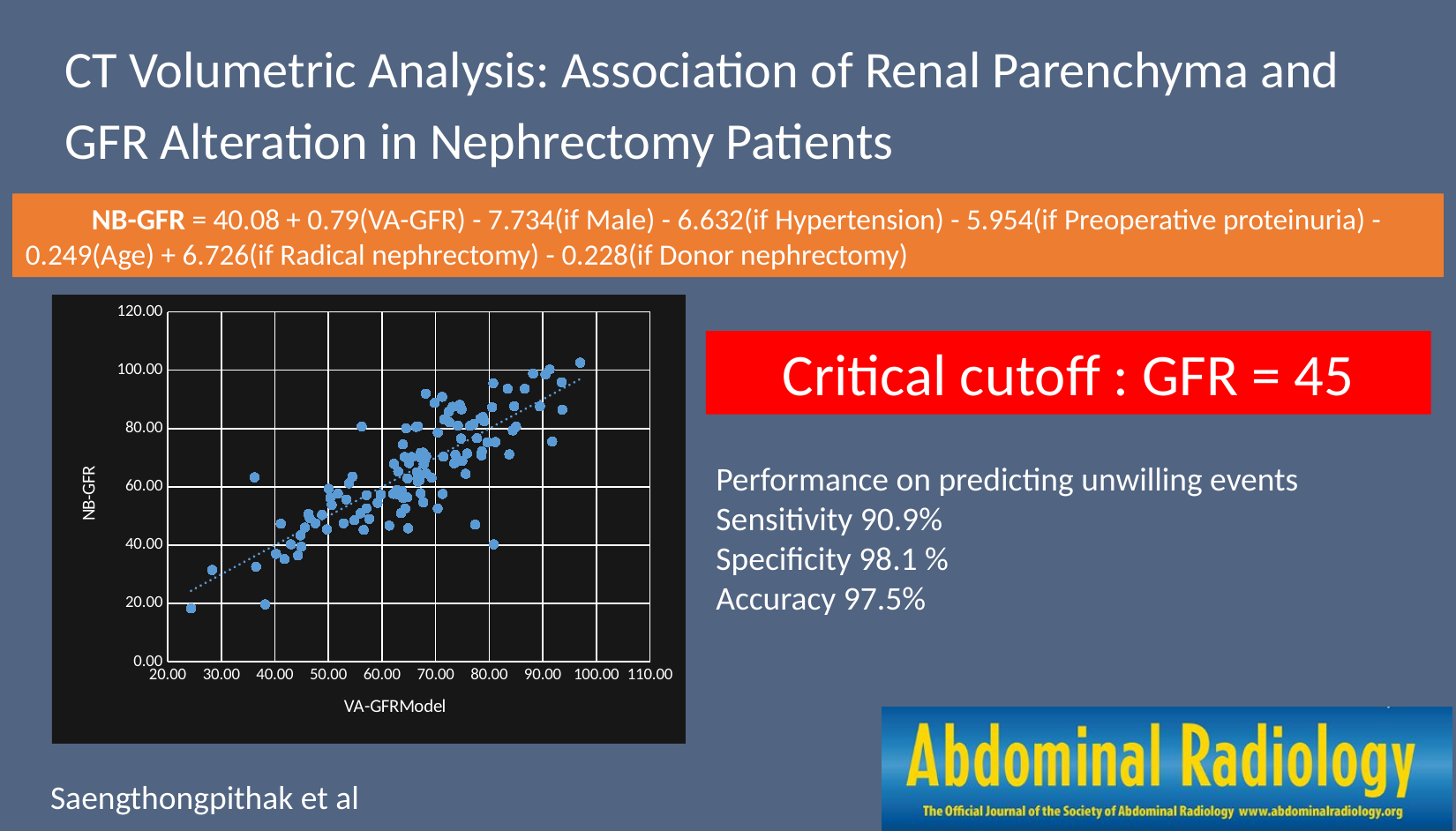

CT Volumetric Analysis: Association of Renal Parenchyma and GFR Alteration in Nephrectomy Patients
NB-GFR = 40.08 + 0.79(VA-GFR) - 7.734(if Male) - 6.632(if Hypertension) - 5.954(if Preoperative proteinuria) - 0.249(Age) + 6.726(if Radical nephrectomy) - 0.228(if Donor nephrectomy)
### Chart:
| Category | |
|---|---|Critical cutoff : GFR = 45
Performance on predicting unwilling events
Sensitivity 90.9%
Specificity 98.1 %
Accuracy 97.5%
Saengthongpithak et al

## Slide 5
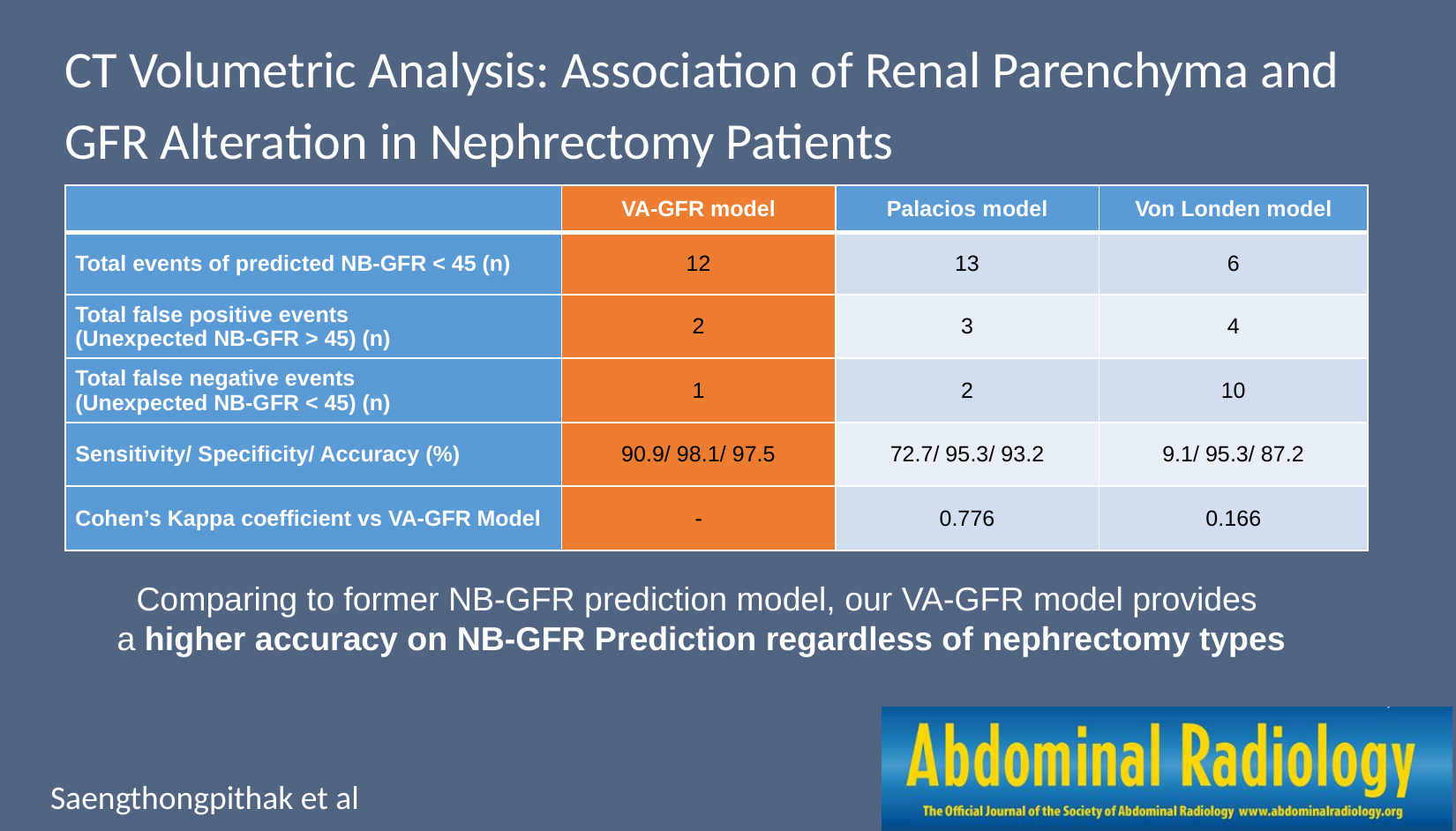

CT Volumetric Analysis: Association of Renal Parenchyma and GFR Alteration in Nephrectomy Patients
| | VA-GFR model | Palacios model | Von Londen model |
| --- | --- | --- | --- |
| Total events of predicted NB-GFR < 45 (n) | 12 | 13 | 6 |
| Total false positive events (Unexpected NB-GFR > 45) (n) | 2 | 3 | 4 |
| Total false negative events (Unexpected NB-GFR < 45) (n) | 1 | 2 | 10 |
| Sensitivity/ Specificity/ Accuracy (%) | 90.9/ 98.1/ 97.5 | 72.7/ 95.3/ 93.2 | 9.1/ 95.3/ 87.2 |
| Cohen’s Kappa coefficient vs VA-GFR Model | - | 0.776 | 0.166 |
Comparing to former NB-GFR prediction model, our VA-GFR model provides
a higher accuracy on NB-GFR Prediction regardless of nephrectomy types
Saengthongpithak et al
