# Supplementary material for: CT volumetric analysis: association of renal parenchyma and GFR alteration in nephrectomy patients
Source: Abdom Radiol (NY). 2024 Dec 6;50(6):2549–57. doi: 10.1007/s00261-024-04693-y (PMC12069147; doi:10.1007/s00261-024-04693-y)
Supplement: Supplementary file 2 — Supplementary Material 2 [file 261_2024_4693_MOESM2_ESM.docx]

Division of Urology

Department of Surgery

Faculty of Medicine, Siriraj Hospital, Mahidol University,

2 Wanglang Road, Bangkok Noi,

Bangkok Noi, Thailand 10700

+66 2419 7000

Editor-in-Chief

Abdominal Radiology

September , 2024

Dear Editor-in-Chief

We are submitting an original research article entitled “CT volumetric analysis : The association of renal parenchyma and GFR alterations in nephrectomy patients” by Wasin Saengthongpithak et al for your considerations in The Abdominal radiology journal publication.

Since the impact of surgically induced CKD on the general population, such as living kidney donors remains unclear, previous studies demonstrate deleterious effects of renal parenchyma loss following treatment of renal cell carcinoma. Partial and radical nephrectomy are the mainstay treatment of RCC, offering promising oncological outcomes. However, the loss of renal function, as indicated a decreased in glomerular filtration rate is inevitable. This decline in GFR not only increases the higher risk of hospitalization and cardiovascular morbidity but also shorter non-cancer-related survival when the new baseline GFR falls below 45.

Our retrospective review study involved a data from patients with renal cell carcinoma and potential renal transplant donors who underwent partial, radical, and donor nephrectomy in siriraj hospital between 2017 – 2021. To demonstrate degree of correlations between volumetric based GFR (VA-GFR) and actual new baseline GFR (NB-GFR) and determine factors affecting NB-GFR decline

We confirm that this work is original and has not been published previously, nor is it currently under consideration for publication elsewhere.

We have no conflicts of interest associated with this publication. There has been no financial support from any funding agency for this work that could have influenced its outcome. As Corresponding Author, I confirm that the manuscript has been read and approved for submission by all the named authors.

We hope you find our manuscript suitable for The Abdominal Radiology journal and looking forward to hearing kind comments from you.

Sincerely,

Thitipat Hansomwong, MD
